# Association of Low-Dose Exposure to Persistent Organic Pollutants with Global DNA Hypomethylation in Healthy Koreans

**DOI:** 10.1289/ehp.0901131

**Published:** 2009-11-06

**Authors:** Keon-Yeop Kim, Dong-Sun Kim, Sung-Kook Lee, In-Kyu Lee, Jung-Ho Kang, Yoon-Seok Chang, David R. Jacobs, Michael Steffes, Duk-Hee Lee

**Affiliations:** 1 Department of Preventative Medicine; 2 Department of Anatomy; 3 Department of Endocrinology, School of Medicine, Kyungpook National University, Daegu, Korea; 4 School of Environmental Science and Engineering, POSTECH, Pohang, Korea; 5 Department of Laboratory Medicine and Pathology, School of Medicine, University of Minnesota, Minneapolis, Minnesota, USA; 6 Division of Epidemiology, School of Public Health, University of Minnesota, Minneapolis, Minnesota, USA; 7 Department of Nutrition, University of Oslo, Oslo, Norway

**Keywords:** epigenetics, hypomethylation, organochlorine pesticides, persistent organic pollutants

## Abstract

**Background:**

Global DNA methylation levels have been reported to be inversely associated with blood levels of persistent organic pollutants (POPs), xenobiotics that accumulate in adipose tissue. Whether these associations extend to a population with much lower concentrations of POPs is not known.

**Objectives:**

This study was performed to examine whether low-dose exposure to POPs was associated with global DNA hypomethylation in Koreans.

**Methods:**

The amount of global DNA hypomethylation was estimated by the percent 5-methyl-cytosine (%5-mC) in *Alu* and LINE-1 assays in 86 apparently healthy Koreans. Among various POPs, organochlorine (OC) pesticides, polychlorinated biphenyls (PCBs), and polybrominated diphenylethers (PBDEs) were measured.

**Results:**

Most OC pesticides were inversely and significantly associated with %5-mC in the *Alu* assay, with correlation coefficients in the range −0.2 to −0.3 after adjusting for age, sex, body mass index, smoking, and alcohol. The strongest OC pesticide associations with %5-mC in the *Alu* assay were observed with oxychlordane, *trans*-nonachlor, and *p,p*′-dichlorodiphenyldichloroethylene. The correlation coefficient of age with %5-mC in the *Alu* assay was −0.24, similar to correlations of OC pesticides with %5-mC in the *Alu* assay. Most PCBs and PBDEs showed nonsignificant inverse trends with %5-mC in the *Alu* assay, but for some PCBs the U-shaped association was significant. On the other hand, POPs were not associated with %5-mC in the LINE-1 assay.

**Conclusions:**

We found that low-dose exposure to POPs, in particular OC pesticides, was associated with global DNA hypomethylation in apparently healthy Koreans.

Recent cross-sectional studies have reported that serum concentrations of persistent organic pollutants (POPs) are associated with a variety of obesity-related diseases including type 2 diabetes and metabolic syndrome in the U.S. general population with only low-dose exposure to POPs (Lee et al. 2006a, 2006b, 2007). In particular, a well-established association between obesity and type 2 diabetes became stronger as serum concentrations of POPs increased, but obesity was not associated with type 2 diabetes among subjects with very low levels of POPs (Lee et al. 2006b).

Even though underlying mechanisms are largely unknown, epigenetic mechanisms have been suggested as a possible explanation for the associations between POPs and various diseases (Porta 2006). DNA methylation constitutes an essential epigenetic characteristic that influences a wide variety of biological processes including gene expression, chromosomal stability, imprinting, and cellular differentiation (Bernstein et al. 2007). Disturbance of epigenetic modulations is considered to be a key mechanism in many diseases (Ozanne and Constancia 2007). In addition, global DNA hypomethylation is thought to constitute an early event in some cancers and can progressively occur and accumulate during aging (Wilson et al. 2007).

Supporting this idea, a recent study among Greenland Inuit has reported that global DNA methylation levels are inversely associated with blood levels of several POPs (Rusiecki et al. 2008). However, as the exposure levels in Greenland Inuit are much higher than those in the U.S. general population, it is unclear whether the findings observed in Greenland Inuit apply to other populations with very low levels of POPs.

This study was performed to examine whether the exposure to low-dose POPs is associated with global DNA hypomethylation of peripheral blood leukocytes in the apparently healthy Korean population. Global DNA hypomethylation was measured by percentage of global genomic DNA methylation, percent 5-methyl-cytosine (%5-mC) in *Alu* and long interspersed nucleotide element (LINE-1) assays.

## Materials and Methods

### Study subjects

A community-based health survey was performed from June 2006 to December 2006 in Uljin county, South Korea. This county is located on the shore of the East Sea, and the majority of the population is involved in fishery and agriculture. Residents ≥ 40 years of age were invited to participate in a survey through a local newspaper and the board of a community health service center and a local hospital. Study subjects were 86 apparently healthy adults who were randomly selected from 1,007 participants as controls in ongoing case–control studies on associations of POPs with diabetes or metabolic syndrome. This study was conducted with approval from the Institutional Review Board at the Kyungpook National University Hospital. Additionally, informed written consent was obtained from all subjects before they participated in the study.

Demographic information and lifestyle factors were determined for all participants by trained interviewers using a standardized questionnaire in a face-to-face interview. Height and body weight were measured using standard methods in light clothes. Body mass index (BMI) was calculated as weight divided by height squared (kilograms per square meter). Systolic and diastolic blood pressures were measured in the sitting position after a 5-min rest. Three blood pressure readings were obtained at 1-min intervals and were averaged.

Blood samples were obtained by venipuncture after overnight fasting, and the serum samples were separated by centrifugation and transferred to contamination-free bottles with Teflon-coated caps. All samples were kept frozen at −70°C until analysis. Fasting glucose, triglycerides, and high-density lipoprotein (HDL) cholesterol were determined by enzymatic methods using ADVIA 1650 (Bayer Inc., Tarrytown, NY, USA).

### POPs measurement

POPs were analyzed in 2 mL serum at the laboratory of the School of Environmental Science and Engineering, POSTECH (Pohang, Korea) using isotope dilution method with gas chromatography-high resolution mass spectrometry (GC-HRMS). Briefly, the serum samples were spiked with isotopically labeled standards of organochlorine (OC) pesticides (ES-5349, Cambridge Isotope Labs, Andover, MA, USA), polychlorinated biphenyls (PCBs) (P48M-ES, Wellington Laboratories Inc., Wellington, Ontario, Canada), and polybrominated diphenylethers (PBDEs) (MBDE-MXFS, Wellington Laboratories). Samples were then extracted on C18 solid-phase extraction (SPE) cartridges (Waters, Milford, MA, USA). The eluate was applied to a silica gel/florisil SPE cartridge (Waters), then eluted with hexane followed by dichloromethane/hexane (1:1 vol/vol). Custom-made acid silica SPE cartridges were used for additional cleanup of PBDEs analysis. The extracts were evaporated using HyperVap (IEC, Seoul, Korea) and transferred to vials. The vials were stored at −70°C until analysis by GC-HRMS. GC-HRMS measurements were performed on a JMS-800D instrument (JEOL, Tokyo, Japan) interfaced with a 6890N gas chromatograph (Agilent Technologies, Santa Clara, CA, USA). PBDEs were measured only among 50 study subjects.

Total lipids were calculated using the short formula (Phillips et al. 1989): total lipids (mg/dL) = 2.27 × total cholesterol + triglycerides + 62.3. We used lipid-standardized concentrations of POPs by dividing wet concentrations of POPs by total lipids throughout the analyses. Among 22 OC pesticides, 34 PCBs, and 8 PBDEs measured in this study, we selected 9 OC pesticides, 17 PCBs, and 2 PBDEs for which at least 80% of study subjects had concentrations more than the limit of detection (LOD).

### Blood DNA extraction and bisulfite treatment

Genomic DNA was extracted from whole-blood samples using a Gentra Puregene Blood kit (Qiagen, Valencia, CA, USA). One microgram DNA was bisulfite-modified by using the EZ DNA Methylation-Gold Kit (Zymo Research, Orange, CA, USA) according to the manufacturer’s protocol. Final elution was performed with 30 μL M-Elution Buffer (Zymo Research) and was stored at −70°C until use.

We used a built-in control to verify bisulfite conversion efficiency by pyrosequencing: we used a C outside a CG site. After bisulfite treatment, the conversion of this C into T is expected to be 100%. It is possible to insert a C/T single-nucleotide polymorphism into the sequence to be analyzed that will result in a 100% T if conversion is efficient. Built-in analysis of non-CpG cytosine residues provided an internal control for the completeness of bisulfate treatment.

### Assay of Alu and LINE-1 methylation

Methylation analyses of *Alu* and LINE-1 were quantitatively performed on the bisulfite-treated DNA by using pyrosequencing with the same PCR primers and conditions as previously described (Bollati et al. 2007; England and Pettersson 2005). In brief, the bisulfite-treated samples (50 ng) were amplified with a biotin-labeled primer through PCR, which enables the conversion of the PCR product to a single-stranded DNA template suitable for pyrosequencing. Confirmation of the quality of the PCR products and freedom from contamination was established on 2% agarose gels with ethidium bromide staining. After purification of PCR products using Sepharose beads on PyroMark Vacuum Prep Workstation (Qiagen), pyrosequencing was carried out using the PyroMark Q96MD System (Qiagen) according to manufacturer’s protocol, including single-strand binding protein.

The percentage of methylation was expressed for each DNA locus as %5-mC divided by the sum of methylated and unmethylated cytosines. We tested each marker in three replicates and used their average in the statistical analyses. Three controls were included in every pyrosequencing run. One well was filled with water to ensure no contamination, and two wells were filled with CpGenome Universal methylated and unmethylated DNA (Chemicon, Temecula, CA, USA) to evaluate the repeatability of the assay.

### Statistical analyses

Pearson correlation coefficients between log-transformed serum concentrations of POPs with %5-mC in *Alu* and LINE-1 assays were calculated. We presented both crude and adjusted correlation coefficients. Possible confounders were age (years), sex, cigarette smoking (continuous), BMI (continuous), and alcohol consumption (continuous). Crude and adjusted means of %5-mC in *Alu* and LINE-1 assays by quintiles of serum concentrations of POPs were examined by general linear models. As dose–response relationships were not clearly linear, we presented both *p* for trend and *p* for the quadratic term. All data were analyzed using SAS version 9.1 (SAS Institute Inc., Cary, NC, USA).

## Results

Table 1 shows demographic and clinical characteristics of study subjects. Of the 86 study participants, 34 (39.5%) were men and 52 were (60.5%) women. Mean age (range) was 56.2 years (42–69 years). Percent global DNA methylation in the *Alu* and LINE-1 assay were 21.1 ± 0.9 and 76.6 ± 2.9. When we compared percent global DNA methylation by demographic and health behavior variables, age showed a statistically significant inverse association with DNA methylation only in the *Alu* assay (*r* = −0.24, *p* = 0.03), not the LINE-1 assay (Table 2). Percent global DNA methylation in the *Alu* and LINE-1 assay were not statistically different by sex, BMI, smoking status, alcohol-drinking status, or other clinical variables.

Table 3 shows POPs distribution of the study subjects. Compared with the Greenland Inuit (Rusiecki et al. 2008), most POPs were lower in these subjects by several orders of magnitude. However, both beta-hexachlorocyclohexane and *p,p*′-DDT showed similar concentrations between these two groups of study subjects.

Pearson correlation coefficients of various POPs with *Alu* and LINE-1 assays are displayed in Table 4. Most OC pesticides were inversely and significantly associated with %5-mC in the *Alu* assay, with correlation coefficients from about −0.2 to −0.3. Adjustment for age, BMI, cigarette smoking, and alcohol consumption did not change the results. The strongest associations were observed with *p,p*′-dichlorodiphenyldichloroethylene, oxychlordane, and *trans*-nonachlor. Most PCBs also showed inverse associations with the *Alu* assay; however, strength of associations were weaker than the correlations of %5-mC in the *Alu* assay with OC pesticides. Thus, the associations for most PCBs, except PCB153, PCB183, and PCB187, failed to reach statistical significance. PBDEs also showed weak inverse associations with %5-mC in the *Alu* assay. On the other hand, %5-mC in the LINE-1 assay was not associated with POPs.

To further examine the correlation results, we examined adjusted means of *Alu* and LINE-1 assays by quintiles of several selected OC pesticides that showed significant inverse associations (Table 5). Even though *p*-values for trend were more significant than *p*-values for quadratic terms with oxychlordane, *trans*-nonachlor, and *p,p*′-DDE, the inverse associations were more evident with lower ranges of these POPs (1st quintile to 3rd quintile). For POPs in the 4th and 5th quintiles, there were plateaus or slight increases of DNA methylation. Because the correlation analyses in Table 4 could miss true associations if there were nonlinear associations, we again examined all PCBs using general linear models and presented results for several selected PCBs (Table 5). U-shaped associations were highly evident for several PCBs that had nonsignificant linear correlations in Table 4.

## Discussion

In the current study, DNA methylation measured by the *Alu* assay decreased with increasing concentrations of POPs. Among different subclasses of POPs, OC pesticides were most strongly and consistently associated with %5-mC in the *Alu* assay. Even though some congeners of PCBs and PBDEs showed inverse trends, strengths of associations were weaker than those of OC pesticides and most of them failed to reach statistical significance. Importantly, our study extends the findings of Rusiecki et al., in that the serum concentrations of most POPs in the current study subjects were much lower than those in the Greenland Inuit (Rusiecki et al. 2008). Their concentrations were similar to those in the U.S. general population; among them, serum concentrations of POPs were associated with various diseases (Lee et al. 2006a, 2006b, 2007).

It is worthwhile to note that the inverse correlations of OC pesticides with %5-mC in the *Alu* assay were similar in magnitude to the correlation between age and global methylation in this study. Adjustment for age did not change the results. At present, aging is known as the most important determinant of global hypomethylation in the general population (Wilson et al. 2007). In fact, serum concentrations of POPs increase with aging in the general population (Lee et al. 2006a, 2006b, 2007), and the well-known association between aging and DNA global hypomethylation may be partly explained by POPs. For example, as observed in the present study, when serum concentrations of *p,p*′-DDE, oxychlordane, and *trans*-nonachlor were adjusted, the correlation between age and %5-mC in the *Alu* assay became nonsignificant (*r* = −0.17, *p* = 0.12).

This study is relevant to the emerging evidence indicating that exposure to environmental factors such as chemicals may contribute to epigenetic modifications such as altered DNA methylation, which in turn influences the risk of diseases of complex etiology (Feinberg 2007). POPs are of particular concern because they bioaccumulate in adipose tissue throughout life, continuously circulate in the body with blood lipids, and affect various organs (Porta et al. 2008). Among POPs, much attention has focused on aryl hydrocarbon receptor (AhR) agonists such as dioxins. However, the POPs measured in this study do not bind to AhR; hence, the fact that their levels were inversely associated with methylation suggests that mechanisms unrelated to AhR may also be relevant to explain adverse health effects of POPs. In addition, it is worthwhile to note that OC pesticides were the subclass of POPs most strongly and consistently associated with type 2 diabetes and metabolic syndrome in previous studies (Ha et al. 2007; Lee et al. 2006a, 2006b, 2007).

However, %5-mC in the LINE-1 assay was not associated with serum concentrations of POPs. In the Greenland Inuit study (Rusiecki et al. 2008), even though both *Alu* and LINE-1 showed inverse associations with POPs, the results were more marked and statistically significant for the *Alu* assay. As *Alu* and LINE-1 are regulated through different mechanisms (Gonzalgo and Jones 1997) and have different transcription patterns in response to cellular stressors (Li and Schmid 2001), different associations with POPs are possible. In fact, in this study, the inverse association between age and DNA hypomethylation was significant only with the *Alu* assay. Even though %5-mC in the LINE-1 assay showed an inverse association with age, it failed to reach statistical significance. Thus, the *Alu* assay may be a more sensitive method in the general population to detect global DNA hypomethylation in peripheral blood leukocytes than the LINE-1 assay.

Further discussion is warranted of the observation that current background exposure to POPs was associated with global DNA hypomethylation in the general population. In particular, dose–response curves of POPs with methylation were not completely linear. Linear inverse associations were mostly restricted to the lower ranges of background exposure to POPs. In the higher ranges of background exposure to POPs, DNA methylation did not decrease, or even increased slightly, suggesting low-dose effects of POPs. We previously speculated about the possibility of low-dose effects of POPs (Lee et al. 2006a) in interpretation of our previous studies on the associations of POPs with disease outcomes such as diabetes or metabolic syndrome (Lee et al. 2006a, 2006b, 2007). In those studies, although the strength of association tended to increase as the concentrations of POPs increased, the association was much steeper across lower background concentrations of POPs than across higher background concentrations of POPs. In fact, low-dose effects are an important property of endocrine disruptors: strong biological effects at low doses, but weak or no effects at high doses (Welshons et al. 2003).

Even though the POP levels are much higher in the Greenland Inuit than in these Korean study subjects, absolute levels of %5-mC were lower in the Koreans than in the Greenland Inuit. There can be several explanations for this finding. First, this finding could be further evidence of low-dose effects of POPs. Second, the finding may have resulted because our study subjects were, on average, older than the Greenland Inuit. Third, these two populations are likely to be very different in terms of diet or exposures to other xenobiotics, which can affect DNA methylation. Studies in cell culture and animal models have reported that xenobiotics such as methoxychlor, vinclozolin, or arsenic can cause disturbance of DNA methylation patterns (Alworth et al. 2002; Anway et al. 2005; Chen et al. 2004). However, besides the Greenland Inuit study, only one human study has examined associations between arsenic and methylation pattern in Bangladeshi adults who are chronically exposed to arsenic-contaminated drinking water (Pilsner et al. 2007). Contrary to findings from experimental studies, arsenic exposure was positively associated with DNA methylation in human leukocytes.

In this study, cigarette smoking, alcohol consumption, and obesity were not associated with DNA methylation of leukocytes. Previous studies have reported that lifestyle factors such as cigarette smoking, alcohol consumption, or dietary intake were inconsistently associated with the extent of methylation (Cravo and Camilo 2000; Figueiredo et al. 2009; Smith et al. 2007). Their effects on methylation may differ depending on cell type, organ, or stage of disease (Kim 2004). In addition, even though the status of nutrients has been studied as a main determinant of DNA methylation, the effects of folate depletion or supplementation on methylation have not been consistent (Kim 2004).

This study has several limitations. First of all, as this study is cross-sectional, we cannot rule out the possibility that change in the methylation pattern of certain genes involved in the metabolism of xenobiotics can affect serum concentrations of POPs. Second, as the study size was small, we could not examine associations after stratification by sex. If POPs play a role as endocrine disruptors, the associations might be different depending on sex. Third, as people are simultaneously exposed to a variety of POP mixtures (mainly through food), we cannot rule out the possibility that other pollutants correlated with POPs may contribute to these findings. Fourth, we did not measure nutrients involved in methylation cycle in blood or other tissue. The inverse associations between POPs and DNA methylation may be stronger when these nutrients are deficient in body tissue. Finally, we used two repetitive DNA elements, namely, *Alu* and LINE-1 repeats, to estimate global methylation. The global content of 5-methyl cytosine could be more accurately measured by methods like total digestion of genomic DNA to nucleotide or nucleoside levels and further separation by HPLC or by capillary electrophoresis. However, DNA methylation analyses of *Alu* and LINE-1 repetitive elements can serve as a surrogate measure of methylation because of the heavy methylation of repetitive elements (Yang et al. 2004), and these methods are easier to carry out than quantification of total genomic 5-mC in epidemiologic studies.

## Conclusion

In conclusion, we found that background exposure to POPs was associated with global DNA hypomethylation in apparently healthy Koreans. In addition, inverse associations were observed mostly in the lower ranges of background exposure to POPs, rather than the higher ranges.

## Figures and Tables

**Table 1:** General characteristics and %5-mC in *Alu* and LINE-1 markers of study subjects.

Characteristic	Value
Age (years)	56.2 ± 7.0
BMI (kg/m^2^)	22.4 ± 2.5
Systolic blood pressure (mmHg)	116.2 ± 12.8
Diastolic blood pressure (mmHg)	71.4 ± 8.2
Fasting glucose (mg/dL)	88.5 ± 8.3
Triglyceride (mg/dL)	87.3 ± 39.0
HDL cholesterol (mg/dL)	51.4 ± 10.3
*Alu* assay	21.1 ± 0.9
LINE-1 assay	76.6 ± 2.9
Men	39.5%
BMI ≥ 25 kg/m^2^	15.1%
Smoker	25.6%
Drinker	46.5%

Values are mean ± SD or percent.

**Table 2:** Spearman correlation coefficients of age and health behaviors with *Alu* assay and LINE-1 assay.

	*Alu* assay		LINE-1 assay	
Characteristic	Crude	Age adjusted	Crude	Age adjusted
Age	−0.24*	—	−0.11	—
Sex	−0.01	0.02	−0.13	−0.12
BMI	−0.10	−0.14	−0.12	−0.14
Cigarette smoking	−0.01	−0.02	0.02	0.02
Alcohol drinking	0.09	0.02	0.09	0.06
Systolic blood pressure	0.02	0.02	0.14	0.14
Diastolic blood pressure	0.03	0.01	0.15	0.13
Fasting glucose	−0.01	0.04	−0.01	0.01
Triglyceride	0.13	0.16	0.03	0.04
HDL cholesterol	0.01	0.01	−0.10	−0.10

* *p* < 0.05

**Table 3:** Distribution of serum concentrations (nanograms per gram lipid) of POPs.

Compound	Current study subjects							Greenland Inuit^a^ median
	Detection rate (%)	Mean LOD	20%	40%	Median	60%	80%	
β-HCH	100	1.4	20.9	29.4	37.3	44.5	58.0	40.0
HCB	94.2	0.4	3.5	12.0	14.0	16.6	21.5	239.3
Heptachlor epoxide	95.4	0.9	3.1	4.0	5.3	6.9	11.5	
Oxychlordane	86.1	1.7	3.2	6.3	7.3	8.0	12.8	249.0
*trans-*Nonachlor	98.8	4.1	9.2	13.7	17.4	20.9	27.9	
*p,p*′-DDE	100	1.6	168.1	235.6	336.8	393.0	566.1	1268.5
*p,p*′-DDD	83.7	0.8	1.6	3.2	3.7	4.5	6.6	
*p,p*′-DDT	97.7	9.5	11.5	17.4	20.2	23.3	30.9	25.1
Mirex	88.4	0.4	0.7	1.2	1.8	2.0	3.3	38.9
PCB74	96.5	1.0	3.5	5.7	6.5	7.2	10.6	
PCB99	98.8	1.4	4.3	6.6	8.3	10.7	13.9	92.5
PCB105	96.5	0.6	3.0	4.3	5.5	6.6	8.7	14.0
PCB118	100	0.6	15.2	22.9	29.1	33.7	48.5	78.5
PCB138	100	0.4	12.0	19.2	25.3	29.5	41.8	412.9
PCB146	100	0.9	2.8	5.0	6.0	6.5	9.8	
PCB153	98.8	0.4	22.5	37.9	46.7	55.7	85.2	687.1
PCB156	100	0.4	2.4	3.5	4.3	5.1	6.7	
PCB157	82.6	0.4	0.4	0.9	1.1	1.3	1.9	
PCB164	100	0.9	4.9	8.3	9.8	10.5	16.5	
PCB167	94.2	0.9	1.0	1.4	1.8	2.0	3.1	
PCB172	97.7	0.2	0.7	1.0	1.2	1.5	2.3	
PCB177	98.8	0.2	1.0	1.4	1.8	1.9	3.3	
PCB178	100	0.2	1.0	1.6	1.9	2.2	3.2	
PCB180	100	0.7	15.6	21.7	28.0	34.4	52.1	318.4
PCB183	98.8	0.2	1.3	2.0	2.5	3.1	4.5	39.0
PCB187	98.8	0.7	3.9	7.6	8.9	11.7	15.6	140.8
BDE47	98.0	0.8	1.4	2.4	3.1	3.7	6.5	
BDE99	98.0	0.5	0.7	1.4	1.7	2.3	3.4	

a Data from Rusiecki et al. (2008).

**Table 4:** Pearson correlation coefficients of log-transformed serum concentrations of POPs with %5-mC in *Alu* and LINE-1 assays.

	*Alu* assay		LINE-1 assay	
Compound	Crude	Adjusted^a^	Crude	Adjusted^a^
β-HCH	−0.26*	−0.22	−0.05	0.04
HCB	−0.22*	−0.18	−0.05	0.08
Heptachlor epoxide	−0.30**	−0.23*	−0.08	−0.02
Oxychlordane	−0.32**	−0.28*	−0.02	−0.01
*trans-*Nonachlor	−0.25*	−0.28*	−0.03	−0.07
*p,p*′-DDE	−0.27*	−0.29**	−0.03	−0.05
*p,p*′-DDD	−0.19	−0.15	−0.06	−0.04
*p,p*′-DDT	−0.20	−0.22*	0.12	0.11
Mirex	−0.17	−0.15	−0.01	−0.06
PCB74	−0.12	−0.13	−0.13	−0.12
PCB99	−0.18	−0.16	−0.04	−0.06
PCB105	−0.08	−0.12	−0.07	−0.07
PCB118	−0.15	−0.10	−0.09	−0.07
PCB138	−0.17	−0.19	−0.04	−0.08
PCB146	−0.05	−0.06	0.03	−0.02
PCB153	−0.23*	−0.17	−0.03	−0.06
PCB156	−0.06	−0.10	−0.01	−0.07
PCB157	−0.15	−0.15	−0.07	−0.14
PCB164	−0.16	−0.17	0.01	−0.05
PCB167	−0.01	−0.05	0.05	0.03
PCB172	−0.09	−0.12	0.03	−0.07
PCB177	−0.07	−0.14	−0.01	−0.08
PCB178	−0.13	−0.14	0.04	−0.06
PCB180	−0.18	−0.15	−0.01	−0.07
PCB183	−0.21	−0.23*	−0.02	−0.08
PCB187	−0.21*	−0.19	0.01	−0.05
BDE47	−0.04	−0.25*	0.28*	0.11
BDE99	−0.08	−0.12	0.12	−0.07

a Adjusted for age, sex, BMI, cigarette smoking, and alcohol drinking.

* *p* < 0.05,

** *p* < 0.01.

**Table 5:** Means of %5-mC in the *Alu* assay according to quintiles of serum concentrations of several selected POPs.

	Quintile of serum concentrations of POPs						
Compound	Q1	Q2	Q3	Q4	Q5	*p*_trend_	*p*_quadratic_
Oxychlordane							
Model 1^a^	21.7	21.2	21.0	20.6	21.0	< 0.01	0.06
Model 2^b^	21.6	21.1	21.0	20.7	21.0	0.02	0.06
Model 3^c^	21.6	21.1	21.1	20.7	20.9	< 0.01	0.13

*trans-*Nonachlor							
Model 1	21.4	21.5	20.6	20.7	21.1	0.02	0.07
Model 2	21.4	21.4	20.6	20.9	21.1	0.08	0.05
Model 3	21.5	21.5	20.6	20.8	20.9	< 0.01	0.11

*p,p*′-DDE							
Model 1	21.5	21.3	20.8	20.9	20.9	0.04	0.21
Model 2	21.5	21.2	20.7	20.9	21.1	0.10	0.05
Model 3	21.6	21.2	20.7	20.9	21.0	0.04	0.05

PCB105							
Model 1	21.5	21.1	20.7	20.9	21.2	0.23	< 0.01
Model 2	21.4	21.1	20.7	20.9	21.3	0.43	0.01
Model 3	21.5	21.2	20.6	20.9	21.2	0.19	0.01

PCB118							
Model 1	21.5	21.0	20.9	20.7	21.3	0.25	< 0.01
Model 2	21.5	20.9	21.0	20.7	21.3	0.47	0.01
Model 3	21.5	20.9	21.0	20.7	21.3	0.40	0.01

PCB172							
Model 1	21.5	21.2	20.8	20.8	21.2	0.13	0.03
Model 2	21.4	21.2	20.7	20.9	21.2	0.37	0.03
Model 3	21.5	21.2	20.8	20.7	21.1	0.13	0.04

PCB183							
Model 1	21.7	20.8	21.0	20.6	21.2	0.05	< 0.01
Model 2	21.7	20.8	21.1	20.7	21.2	0.14	< 0.01
Model 3	21.9	20.8	21.1	20.5	21.2	0.02	< 0.01

a Model 1: crude.

b Model 2: adjusted for age.

c Model 3: adjusted for age, sex, BMI, cigarette smoking, and alcohol drinking.
